# The practice of procedural pain assessment and management in neonatal intensive care unit in Ethiopia: Cross‐sectional study

**DOI:** 10.1002/hsr2.533

**Published:** 2022-02-17

**Authors:** Emebet Assefa, Mamude Dinkiye, Temesgen Geleta, Temesgen Tantu, Mekete Wondwosen, Dereje Zewdu

**Affiliations:** ^1^ Department of Pediatrics and Child Health, College of Medicine and Health Science Wolkite University Wolkite Ethiopia; ^2^ Department of Pediatrics and Child Health St. Paul's Hospital Millennium Medical College Addis Ababa Ethiopia; ^3^ Department of Public Health St. Paul's Hospital Millennium Medical College Addis Ababa Ethiopia; ^4^ Department of Obstetrics and Gynecology, College of Medicine and Health Science Wolkite University Wolkite Ethiopia; ^5^ Department of Surgery, College of Medicine and Health Science Wolkite University Wolkite Ethiopia; ^6^ Department of Anesthesia, College of Medicine and Health Science Wolkite University Wolkite Ethiopia

**Keywords:** neonates, NICU, painful procedures, procedural pain

## Abstract

**Background and Aims:**

Neonates in intensive care units undergo frequent painful procedures for diagnostic or care‐related purposes. Untreated pain has serious short‐term and long‐term complications. This study aims to evaluate the frequency of painful procedures, pain assessment, and their analgesic management practice among neonates admitted to the NICU.

**Methods:**

The present study is a hospital‐based cross‐sectional study of neonates admitted at level II NICU of St. Paul hospital millennium medical college in Ethiopia between March and August 2019. Data were collected from medical charts of neonates and bedside observation using a checklist. The parameters included were demographic characteristics, types of painful procedures, pain assessment practice, and analgesic intervention provided during painful procedures. Descriptive statistics, Mann‐Whitney *U*‐test, and Kruskal‐Wallis test were used to compare the number of painful procedures and influencing factors. *P*‐value < .05 was considered statistically significant.

**Results:**

Of the 325 neonates included in this study, a median of 4 (3‐7) painful procedures were performed per neonate in the first 24 hours of NICU stay. Heel lance 280 (20.7%) and Venipuncture 249 (18.41%) were the most commonly performed painful procedures. Of the 1352 painful procedures, none of the neonates received any form of analgesic intervention and none of the neonate's pain scores were documented on their medical chart. The higher number of painful procedures were associated with gestational age between 28 and 31 weeks, birth weight less than 1500 g, and use of CPAP respiratory support *P*‐value <.001, <.001, and .0015, respectively.

**Conclusion:**

Painful procedures were frequently performed in NICU without any form of analgesic intervention. Strategies to introduce neonatal pain assessment and their analgesic management for clinical practice are necessary.

**Abbreviations**
CD: cesarean delivery
CPAP: continuous positive airway pressure,
NICU: neonatal intensive care unit
NIPS: neonatal infant pain scale
SVD: spontaneous vaginal delivery

## 1 INTRODUCTION

Neonates admitted to neonatal intensive care units (NICU) undergo multiple diagnostic or care‐related painful procedures during their hospitalizations. In NICU, each neonate experiences an average of 7.5 to 17.3 painful procedures per day in which most of the procedures is ranged from moderate to severe pain.^1^, ^2^, ^3^, ^4^

The most frequently performed painful procedures were heel lance, suctioning, venipuncture, and peripheral IV insertion.^5^, ^6^, ^7^, ^8^, ^9^

Despite the misconception regards to neonatal pain in the past years, scientific evidence indicates that pain pathways are active and functional as early as 25 weeks of gestation.^10^

Under‐treated pain experienced in the early life of the neonatal period can cause physiological and behavioral changes, which may lead to detrimental long‐term consequences of altered pain sensation, neuro‐behavioral changes, and learning disabilities in later life.^11^, ^12^

Prevention of pain and provision of optimal pain control strategies should be an essential part of standard health care in the NICU.^13^, ^14^

Medical knowledge and perception of neonatal pain and analgesic strategies have been improved among healthcare providers; however, the clinical practice remains unchanged.^15^, ^16^

Scientific evidence demonstrates the analgesic efficacy of non‐pharmacological pain relief strategies^17^, ^18^ including breastfeeding, skin‐to‐skin contact, sweet solutions, multisensorial stimulation, and also pharmacological strategies^19^ to control procedural pain in neonates. However, in the neonatal unit of our hospital, the routine painful procedures are performed without any form of pharmacological and non‐pharmacological measures.

In the developed world, despite the recommendations and guidelines based on evidence, procedural pain in neonates continues to be inconsistently assessed and inadequately managed.^20^, ^21^, ^22^ Consequently, in China and sub‐Saharan African countries, painful procedures are performed without any form of pain control measures.^3^, ^8^

In resources limited countries including Ethiopia, neonatal pain management has not been part of neonatal care where the focus has been confined to saving the lives in need.

Furthermore, resource‐scarce to procure analgesic medication, knowledge deficit, and lack of institutional guidelines remains major challenging issues to implement in clinical practice.^23^, ^24^, ^25^ Overcoming these problems is vital as an attempt to improve pain management practice in neonates.

As far as our search, no studies have been done on this major clinical problem in Ethiopia. This prospective cross‐sectional study was conducted to evaluate the practice of procedural pain assessment and analgesic management among neonates admitted to NICU.

## 2 MATERIALS AND METHODS

### 2.1 Study design and participants

A hospital‐based prospective cross‐sectional study was conducted over 6 months between March 2019 and August 2019 in St. Paul's hospital millennium medical college. This study is done as per the declaration of Helsinki ethical principles for medical research involving human subjects' protocol. Ethical clearance was received from the ethical committee of the institution and written informed consents were obtained from the legal guardian or parents. This study is registered at www. Research registry with research registry UIN: researchregistry7419.

**FIGURE 1:**
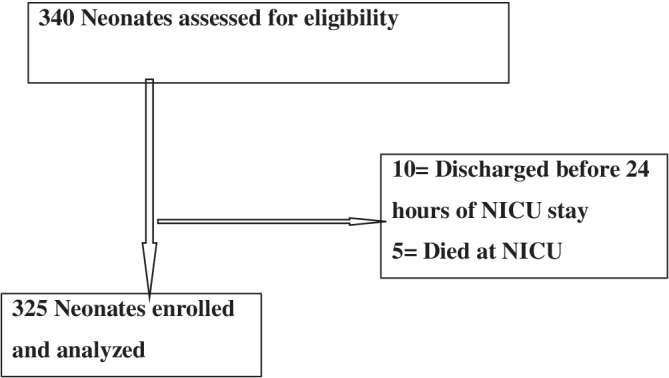
Study flowchart

All neonates admitted to level II NICU with postnatal age of fewer than 7 days and gestational age of >28 weeks and successful attempts of painful procedures were included in this study as shown in Figure 1. Neonates transferred or discharged to another unit before 24 hours of admission and neonates with altered levels of consciousness that affect movement and facial expression were excluded from the study.

### 2.2 Sample size and sampling techniques

The outcome variables of our study were to determine the number of painful procedures, evaluate the practice of pain assessment, and analgesic intervention during the first 24 hours of NICU admission. The sample size was calculated by using the single population proportion formula. The following assumptions were used to compute the sample size; the prevalence of procedural pain (69.6%) was taken from the preliminary data conducted in Netherlands^22^ with a 95% confidence interval, a 5% margin of error, and a 5% attrition rate, the total sample size was 340 study participants. Based on the review of the logbook and registration system of neonates admitted to NICU in the previous year, an average of 690 neonates in 6 months were admitted to this unit.

A systematic random sampling technique was used to select study participants. The sampling interval k was calculated to be 2 using the formula: k = N/n (690/340); where n = total sample size, N = population per 6 months. From all neonates who fulfilled the inclusion criteria, the samples were selected skipping every two intervals. The first participant was selected by lottery method from the first admission day with every two intervals.

### 2.3 Data collection techniques

Data were collected from medical charts and checklists developed based on previous literature. Before data collection, training of 3 days was offered for data collectors regards to overall data collection process including NIPS observation and inter‐rater reliability were measured.

The research team consisted of trained general practitioners, NICU nurses, and pediatricians who compiled a checklist containing all types of daily performed painful procedures. The designed checklist included a list of 11 different procedures routinely performed and considered painful by the team, as shown in Table 1.

**TABLE 1:** Number and type of painful procedures among study participants

Procedures	Frequency	Percentage
Heel stick	280	20.71%
Venipuncture	249	18.41%
Intravenous insertion	217	16%
Intravenous injection	189	13.98%
Intramuscular injection	140	10.35%
Nasogastric tube insertion	111	8.21%
Nasal CPAP insertion	95	7%
Lumbar puncture	28	2.07%
Femoral venous puncture	21	1.55%
Arterial puncture	14	1.12%
Chest tube drainage	8	0.6
Total	1352	100%

Data of demographic characteristics and primary admission diagnosis were collected immediately when the sick neonates were admitted to the NICU from their medical charts.

Frequency and type of painful procedures, NIPS score, and analgesic intervention were collected by bedside observation in real time. The data collection was continued for the first 24 hours of NICU admission.

Neonatal infant pain scale (NIPS) was used to evaluate the intensity of pain on each procedure for 1 minute to fully assess each indicator and graded from 0 to 7. This pain scale assesses six behavioral indicators (facial expression, cry, breathing pattern, arms, legs, and state of arousal) in response to painful procedures in preterm and term neonates and has shown good inter‐observer reliability.^26^, ^27^

The collected data were cross‐checked by the principal investigator to ensure accuracy and completeness.

### 2.4 Statistical analysis

Data were coded and entered into SPSS software for Windows (version 26, SPSS Inc., Chicago, Illinois) was used for the statistical analysis of the data. The Shapiro‐Wilk test was used to test the normality of the distribution of data.

Nonparametric continuous variables were expressed as median and interquartile range (IQR), and categorical variables as frequency and percentage (%, n/N).

Influencing factors and the number of painful procedures were compared between groups using unpaired *t*‐test and analysis of variance for parametric data, the Mann‐Whitney *U*‐test, or the Kruskal‐Wallis test for nonparametric data. *P*‐value < .05 on the two‐sided test was considered to be statistically significant.

### 2.5 Operational definitions

**Painful procedure**: Any care‐related or diagnostic interventions that have some degree of pain associated with it.

**Skin breaking and non‐skin breakings**: Procedures were classified as skin breaking if they involved a penetration to the skin (eg, heel stick, injections) and non‐skin breaking if they did not penetrate the skin surface (eg, gastric tube and nasal cannula insertion).^28^

**Procedural pain**: Pain related to either diagnostic or care‐related procedures.

**The severity of pain**: Based on the neonatal infant pain scale (NIPS), the pain intensity was rated as mild to no pain (NIPS:0‐2), mild to moderate (NIPS: 3‐4), and moderate to severe (NRS: >4).

**Average pain score**: The pain score calculated from all the documented procedural pain scores for the individual neonate during the day of study.

**Analgesic**: A medication used for pain relief.

## 3 RESULTS

During the study period between March 1, 2019, and August 30, 2019, of 340 selected sick neonates admitted to NICU, 325 neonates were included in the study (Figure 1).

### 3.1 Number and types of painful procedures

A total of 1352 (1146 skin breaking and 206 non‐skin breakings) painful procedures were performed over a first 24‐hour period of NICU stay with an average of mean = 4.2, SD = 1.05 per neonates per day. A total of 11 different painful procedures were performed, among which the most common was heel stick (20.71%, n = 280/1325) followed by venipuncture (18.41%, n = 249/1325), Iv‐line insertion (16%, n = 217/1325), Iv injection (13.98%, n = 189/1325), IM injection (10.35%, n = 140/1325), nasal gastric tube insertion (8.21%, n = 111/1325), and (7%, n = 95/1325) nasal CPAP insertion (Table 1).

### 3.2 Demographic characteristics

Among 325 neonates, 54.2% (n = 176/325) were male with a male to female ratio of 1.2:1. The majority (82.2%, n = 267/325) of the neonates who were admitted within 24 hours postnatal age (17.2%, n = 58/325) were between 2 and 7 days. More than two‐thirds of the neonates, 228 (73.8%, n = 228/325), were delivered at term, followed by late preterm (18.9%) and early preterm (8%). Regards to gestational age, more than two‐thirds (71.2%, n = 223/325) were >37 weeks, (20.2%, n = 66/325) between 32 and 36 weeks, (7.4%, n = 24/325), were between 28 and 31 weeks, and the rest (3.7%, n = 12/325) were with unknown gestational age (Table 2).

**TABLE 2:** Demographic characteristics and primary diagnosis of study participants

Variables	Category	Frequency	Percentage
Gender	Male	176	54.2%
	Female	149	45.8%
Gestational age (weeks)	28‐31	24	7.4%
	32‐36	66	20.2%
	>37	223	71.2%
	Unknown	12	3.7
Postnatal age	<24 hours	267	82.2
	2‐7 days	58	17.8
Birthweight (grams)	<1500	34	10.5%
	1500‐2500	81	24.91%
	>2500	210	64.5%
Mode of delivery	SVD	196	60.3
	CD	110	33.8
	Instrumental delivery	19	5.8
Primary admission diagnosis	Respiratory distress	108	33.2%
	Hypothermia	68	20.9%
	Neonatal sepsis	62	19.1%
	NHB	52	16%
	Hypoglycemia	23	7.1%
	Others	12	3.7%

With regards to birth weight, the majority (64.5%, n = 210/325) of neonates were greater than 2500 g, followed by 24.91% (n = 81/325) between 1500 and 2500 g, and 10.5% (n = 34/325) less than 1500 g. Spontaneous vaginal delivery was the commonest mode of delivery, which accounted for 60.3% (n = 196/325), followed by cesarean delivery (33.8%, n = 110/325) and instrumental delivery (5.8%, n = 19/325). The primary causes of admission diagnosis were respiratory distress in 33.2% (n = 108/325) followed by 20.9% (n = 68/325) hypothermia, 19.1% (n = 62/325) neonatal sepsis, 16% (n = 52/325) neonatal hyper‐bilirubinemia, and 7.1% (n = 23/325) hypoglycemia (Table 2).

### 3.3 Pain assessment and their analgesic management practice in NICU

There were no validated protocols or guidelines for routine neonatal pain assessment and analgesic management in the study area during the study period. As a result, pain scores were not documented on the medical charts of neonates who were exposed to painful procedures. Similarly, none of the neonates received any form of analgesic intervention before and during painful procedures. The average mean NIPS score observed in this study was 5.08 ± 1.23. All procedures included in this study were painful in which the majority, 64.3%, of procedures ranged from moderate to severe pain intensity, while the rest 35.7% of procedures were associated with mild to moderate pain.

### 3.4 Factors influencing the number of painful procedures

Factors influencing the number of painful procedures performed during the first day of admission are shown in Table 3.

**TABLE 3:** Factors influencing the number of painful procedures

Variables		Number of procedures			
		Total	Median (IQR)	Z or X^2^	*P*‐value
Gender	Male	718	4 (2)	1.406	.165
	Female	607	4 (2)		
Birth weight (grams)	<1500	191	6 (1)	52.231	<.001
	1500‐2500	309	4 (2)		
	>2500	825	4 (2)		
Gestational age (weeks)	28‐31	126	5 (2)	15.185	<.001
	32‐36	276	4 (2)		
	>37	923	4 (1)		
Nasal CPAP	Yes	414	4 (3)	2.401	.016
	No	911	4 (2)		

*Note*: Z was obtained from Mann‐Whitney *U*‐test, and X^2^ were obtained from Kruskal‐Wallis test.

Abbreviation: IQR, Interquartile range.

In terms of gestational age, the median number of painful procedures performed on neonates between 28 and 31 weeks of gestation was significantly higher compared with the median value of neonates between 32 and 36 weeks of gestation and older than 37 weeks of gestation with a *P*‐value <.001. The median number of painful procedures performed according to birth weight was significantly lower on neonates, weighing <1500 g compared with neonates between 1500 and 2500 g and greater than 2500 g *P*‐value <.001, whereas the median value of neonates weighing 1500 to 2500 g and >2500 g demonstrated an insignificant difference *P*‐value = .215.

The median number of painful procedures performed on neonates who received oxygen support via CPAP was higher compared with other *P*‐value = .015. There was no significant difference observed between gender *P*‐value = .165. However, the median value between 32 and 36 weeks and older than 37 weeks of gestation demonstrate an insignificant difference *P*‐value = .762.

## 4 DISCUSSION

To the best of our knowledge, this is the first prospective cross‐sectional study to evaluate the number of procedural pain experiences in neonates admitted to NICU in Ethiopia. This study has revealed that sick neonates admitted to NICU subjected to frequent painful procedures during the first 24 hours of their hospitalization.

Three hundred twenty‐five neonates experienced a total of 1325 skin breaking and non‐skin breaking painful procedures, corresponding to an average of 4.2 per day in each neonate in the first 24 hours of NICU admission, which is similar to four procedures per day, studies done in Kenya.^3^ Inconsistent with our findings, previous studies conducted in different countries reported that each neonate experienced an average of 7 to 13 painful procedures per day.^2^, ^4^, ^6^, ^9^ Another systemic review of observational study^1^ also reported an average of 7.5 to 17.3 per neonate per day.

This discrepancy might be related to the fact that this study was conducted in a level II NICU setting in which relatively lower intervention is required. Secondly, the large majority of neonates were >37 weeks of GA and only 7% were <31 weeks of GA.in our study. Moreover, the number of admitted sick neonates is too high in comparison with the limited number of nurses working in the unit, and the types of painful procedures performed are limited in comparison with previous studies.

In agreement with other studies,^4^, ^5^, ^7^, ^29^ the present findings also show that heel sticks (20.7%) were the most commonly performed painful procedure. Conversely, other studies^2^, ^9^ found suctioning was the most frequently performed procedure. This discrepancy could be explained by mechanically ventilated and a higher number of preterm neonates are included in their studies.

With regards to objective pain assessment and pain management practice, none of the procedures pain scores were documented on the medical chart and all of the painful procedures had been performed without any form of pharmacologic and non‐pharmacological intervention. Unlike other studies, this result is inconsistent with reports from high‐income countries^20^, ^21^, ^22^ in which neonates received some form of analgesic intervention for approximately one‐third to half of the painful procedures with wide variation across the countries, however similar to other resource‐limited settings.^3^, ^7^, ^8^

In resource‐limited settings like Ethiopia, a relatively cheap, accessible, and effective non‐pharmacological intervention like breastfeeding, sugar‐containing solutions, and no nutritive sucking during painful procedures should be included in guidelines and implemented in routine clinical practice. In addition, healthcare providers working in the neonatal unit should be offered training on neonatal pain assessment and management to improve the standard of care.

In terms of factors influencing the number of painful procedures, previous studies observed that preterm infants at the lowest gestational age,^2^, ^4^, ^5^, ^8^ very low birth weight,^2^ and neonates receiving CPAP support^3^, ^4^, ^6^ were exposed to a higher number of painful procedures. Similarly, despite GA and birth weight being strongly correlated, the present study observed that preterm neonates between 28 and 31 weeks of gestation, birth weight <1500 g, and neonates who received CPAP support were exposed to a higher number of painful procedures compared with others. In disagreement with our findings, other studies found no statistically significant difference between the number of painful procedures as compared with gestational age^6^, ^7^, ^9^ and birth weight.^7^ The variation of the statistical analysis method used in their study and ours to determine the association between independent and outcome variables could explain the observed difference.

### 4.1 Limitation of the study

Data were collected in a specified single‐centered teaching hospital, so the findings may not conclude the general features of other NICU centers in Ethiopia. Secondly, a limited number of procedures and higher numbers of skin‐breaking procedures are included in this study. Despite multiple attempts being required, we only included successful attempts, which may underestimate the magnitude of pain.

Further studies involving multicenter hospitals and all routine procedures including unsuccessful attempts are necessary.

### 4.2 Strength of the study

We used a relatively larger sample size and prospective study design.

## 5 CONCLUSION

Our study observed that neonates admitted to NICU were exposed to frequent painful procedures during the first 24 hours of hospitalization. Heel lance was the most commonly performed procedure. Preterm neonates between 28 and 31 weeks of gestational age, birth weight less than 1500 g, and those who require respiratory support experienced more frequent painful procedures. None of the procedures are accompanied by any form of analgesia intervention. We hope that our findings in the practice of pain assessment and management in neonatal pain can bring attention to this clinical activity. Based on our results, we recommend implementing the guidelines of neonatal pain assessment and management in neonatal care centers.

## FUNDING

The author(s) received no financial support for the research, authorship, and/or publication of this article.

## CONFLICT OF INTEREST

The authors declare that the research was conducted in the absence of any commercial or financial relationships that could be construed as a potential conflict of interest.

## AUTHOR CONTRIBUTION

Conceptualization: Emebet Assefa.

Formal Analysis: Mamude Dinkiye, Temesgen Geleta.

Funding Acquisition: Mekete Wondwosen, Temesgen Tantu.

Writing—Original Draft: Emebet Assefa.

Writing—Review, and Editing: Dereje Zewdu.

All authors have read and approved the final version of the manuscript.

Dereje Zewdu had full access to all of the data in this study and takes complete responsibility for the integrity of the data and the accuracy of the data analysis.

## TRANSPARENCY STATEMENT

Dereje Zewdu affirms that this manuscript is an honest, accurate, and transparent account of the study being reported; that no important aspects of the study have been omitted; and that any discrepancies from the study as planned (and, if relevant, registered) have been explained.

## Data Availability

Data are available from the corresponding author upon reasonable request.
